# Evaluations of erectile dysfunction before and after on-pump coronary artery bypass graft surgery

**Published:** 2014

**Authors:** Shervin ZiabakhshTabary, Farzad Mokhtari-Esbuie, Mehran Fazli

**Affiliations:** 1 Cardiac Surgery Department , Fatemeh Zahra Hospital, Mazandaran University of Medical Sciences, Sari, Iran.; 2 Mazandaran University of Medical Sciences, Sari, Iran.; 3 Student Research Committee, Mazandaran University of Medical Sciences, Sari, Iran.

**Keywords:** Erectile dysfunction, Coronary artery bypass graft surgery, IIEF-5

## Abstract

***Background: ***Erectile dysfunction (ED) is common in cardiovascular disease (CVD) and indicates a complication of coronary artery bypass graft surgery (CABG). This study was designed to evaluate the status of erectile dysfunction in patients with CAD before and after CABG surgery.

***Methods:*** We designed a prospective cohort study on male patients candidates for elective on-pump CABG between June 2006 to October 2012 in 3 hospitals in Sari, Iran (Fatemeh Zahra, Shafa and Nime-Shaban Hospitals). Patient’s primary data were collected based on the International Index of Erectile Function questionnaire (IIEF-5) at baseline 6 months after surgery. The severity of dysfunction was categorized as, normal, mild to moderate and moderate to severe based on IIEF score.

***Results:*** Four hundred twenty-six male patients with mean age of 58.69±12.49 years participated in this study. 166 patients had DM (38.96%) 230 patients had hypertension (54%). 307 patients had three-vessel impairment (72.07%) and 119 patients with one or two vessel impairment (27.93%). The 15.02%, 18.07%, 23%, 31.92% and 11.97%, at baseline changed to 16.90%, 19.95%, 23.71%, 32.22% and 8.21%, at endpoint (P=0.01). Also, the mean±SD of IIEF-5 score before and after surgery was 13.95±5.44 and 14.20±5.69, respectively (P=0.001).

***Conclusion: *** The result of our study shows that on-pump CABG surgery has a positive effect on the improvement of erectile dysfunction 6 months after surgery.

Normal sexual functioning plays an important role in happiness and emotional health and sexual dysfunction may have a negative effect on the quality of interpersonal relationships, depression, stress, loneliness condition and even can lead to losing jobs and friends (1). Erectile dysfunction (ED) is defined as the consistent inability to reach and maintain an erection satisfactory for sexual activity (2). This condition involves about 52% of male adults between the ages of 40 and 70 years in the USA and about 322 million men throughout the world (3, 4). Some of the studies show that ED can be related to age, atherosclerosis risk factors, and coronary artery disease (CAD) (4–7). The prevalence of ED in male patients with CAD was reported between 46% and 75% (8-9). Other than vascular disease and endothelial dysfunction in CAD patients, some of the drugs such as beta-blockers and lipid lowering drugs show that they can d increase sexual dysfunction in men and women (10). One of the most common cardiac diseases is coronary artery disease and it is considered the leading cause of morbidity and mortality in the entire world (11-15).

Also, one of the choice treatments for CAD patients is coronary artery bypass grafting surgery (16). Some studies reported that erectile dysfunction can be a postoperative complication of CABG surgery (1, 9, 11, 14). On the basis of these data, we designed this study to evaluate erectile dysfunction in patients with CAD before and after CABG surgery.

## Methods

We designed a prospective cohort study for male patient candidates for elective on-pump CABG between June 2006 to October 2012 in 3 hospitals in Sari, Iran (Fatemeh Zahra, Shafa and Nime-Shaban Hospitals). The inclusion criteria consisted of patients between 40 to 70 years old with CAD confirmed by angiography. Our exclusion criteria included: congestive heart failure, liver disease (ALT or AST level >2 fold of normal range), renal impairment (Cr > 1.5 mg/dl), drug consumption for erectile dysfunction prior to the study, systemic or venous thromboembolism at least 3 months ago, stroke or acute coronary syndrome, active inflammatory disease, infection disease, malignant disease, history of myocardial infarction in the past 6 months, cardiac valvular disease and patients with hormone replacement therapy. Based on our inclusion and exclusion criteria, about 950 patients were able to participate in our study, but only 460 patients were willing to participate in the study and filled out the agreement form.

**Operative procedure: **All patients underwent same method, standardized generalized anesthesia and surgical techniques. All were on-pump CABG and we used antegrade cardioplagic solution via a catheter through aorta every 20 minutes. We used cold saline solution to cool the heart locally. The patient’s body temperature was reduced to 31^C^. In all the patients, we used the left internal mammary artery (LIMA) for the bypass of the left anterior descending (LAD) coronary artery stenosis and saphenous vein graft (SVG) for other coronaries. Proximal of all SVGs were anastomosed on ascending aorta. All surgeries were performed by one surgeon (24). 

**Erectile function evaluation and other data collection: **We used the international index of erectile function questionnaire (IIEF-5) to categorize erectile function. For this purpose, IIEF-5 was filled by one expert examiner before surgery and 6 months after for all the patients. This score questionnaire measured ED severity using five questions, the questions get scores from 1 (high problem) to 5 (no problem) points. Minimum IIEF-5 score show the worst condition. This score questionnaire description included 22-25; no ED, 21- 17; mild ED, 16-12; mild to moderate ED, 11-8; moderate ED and 5-7; severe ED (11). Other variables that were assessed before surgery included: age, history of diabetes mellitus (DM), hypertension (HTN), beta blocker therapy, ACE inhibitor therapy, number of impaired vessels, ejection fraction (EF), blood urea nitrogen (BUN), creatinine (Cr), aspartate aminotransferase (AST) and alanine aminotransferase (ALT). 

**Statistical Analysis: **The data were analyzed by SPSS Version 16. We used mean, standard deviation, and percentage when appropriate for the patient’s characteristic description. Also, we used chi-square test for the comparison of categorical variables and student t-test for continuous variables. P-values under 0.05 were considered statistically significant.

## Results

Finally, four hundred and twenty-six patients were analyzed. The mean age was 58.69±12.49 years. Based on history, 166 patients had DM (38.96%) and 230 patients had HTN (54%). Also, 213 patients (50%) had history of angiotensin converting enzyme inhibitor (ACE-I) use or angiotensin receptor blocker (ARB) use and 205 patients had history of beta-blocker (48.12%) medication. The results of the patient’s angiography showed 307 patients had three- vessel impairment (72.07%) and 119 patients with one or two-vessel impairment (27.93%). All the other patients’ primary data were shown in table 1. 

**Table1:** Patient's demographic and primary data

Age	58.69± 12.49
Diabetes mellitus	166 (38.96%)
Hypertension	230 (54%)
ACE-Inhibitor	213 (50%)
Beta-blocker	205 (48.12%)
One or two vessel disease	119 (27.93%)
Three or more vessel disease	307 (72.07%)
Ejection fraction	51.73±10.08
BUN	19.02±7.20
Cr	0.91±0.25
AST	17.71±7.15
ALT	19.02±8.64

At the end of the study period, the severity of 3D changed from 15.02%, 18.07%, 23%, 31.92% and 11.97% to in 16.90%, 19.95%, 23.71%, 32.22% and 8.21%, respectively (P=0.01). Also, the mean±SD of IIEF-5 score before and after surgery was 13.95±5.44 and 14.20±5.69, respectively (P=0.001) (table2). 

**Table 2:** International index of Erectile Function score before and 6 month after surgery

**College**	**Before** **CABG**	**6 month after** ** CABG**
Normal (22-25)	64 (15.02)	72 (16.90)
Mild ED(17-21)	77 (18.07)	85 (19.95)
Mild to moderate(12-16)	98 (23)	101 (23.71)
Moderate(8-11)	136 (31.92)	133 (31.22)
Severe (5-7)	51 (11.97)	35 (8.21)
IIEF± SD	13.95±5.44	14.20±5.69

P=0.001

ED: erectile dysfunction, IIEF: international index of Erectile Function, SD: standard deviation, CABG: Coronary artery bypass graft.

## Discussion

This study demonstrated a significant improvement of ED in patients with CAD after GABG surgery. However, ED has persisted in the surgical proportion of patients after surgery. While, the existence of erectile dysfunction in CAD patients and some of the etiology such as endothelial damage and atherosclerosis are clearly known, but the effect of CABG surgery and its impact on erectile dysfunction is controversial (17). Some authors believe that CABG surgery with cardiopulmonary bypass (CPB) may have adverse effects on endothelial functions (by reducing the ability of endothelial cells to build and release nitric oxide) and this may lead to the increased risk of the postoperative complications (17, 18). Other authors believe that this method has excellent results without impairment in neurocognitive outcome and quality of life and even CABG with this technique can improve sexual function in patients with CAD (17, 19, 20). 

In our study, the mean IIEF-5 score increased significantly 6 months after surgery. Indicating delayed beneficial effect of surgery on ED which is consistent with some previous studies (17, 19-22) delayed recovery time in ED may be attributed to greater tissue perfusion and improvement of penile erectile function after revascularization (17, 22). Neverthless, improvement in erectile function was not obvious in a number of studies despite improvement in score. In these studies, erectile function decreased or exacerbated after surgery, which has been attributed to some confounding risk factors that may mix up with the results (20, 21, 23). Therefore, in this study we tried to exclude those factors and this may lead to significant improvement in erectile function in our evaluation.

Based on IIEF-5 score, we have more changes and improvements in severe ED patients while the other categories, no significant changes were seen. This result means that CABG surgery has powerful effects on erectile dysfunction improvement especially in severe cases. 

In conclusion, the result of our study shows on-pump CABG surgery has a positive effect on the improvement of erectile dysfunction in 6 months after surgery. Improvement of erectile dysfunction in severe types of dysfunction was more obvious.

## Notes

**Funding:** This project was supported by the Vice-Chancellery for Research of Mazandaran University of Medical Sciences.
